# Subcritical Water Hydrolysis Effectively Reduces the *In Vitro* Seeding Activity of PrP^Sc^ but Fails to Inactivate the Infectivity of Bovine Spongiform Encephalopathy Prions

**DOI:** 10.1371/journal.pone.0144761

**Published:** 2015-12-16

**Authors:** Yuichi Murayama, Miyako Yoshioka, Hiroyuki Okada, Eri Takata, Kentaro Masujin, Yoshifumi Iwamaru, Noriko Shimozaki, Tomoaki Yamamura, Takashi Yokoyama, Shirou Mohri, Yuji Tsutsumi

**Affiliations:** 1 Influenza/Prion Disease Research Center, National Institute of Animal Health, Tsukuba, Ibaraki, Japan; 2 Research Area of Pathology and Pathophysiology, National Institute of Animal Health, Tsukuba, Ibaraki, Japan; 3 Department of Agro-environmental Sciences, Faculty of Agriculture, Kyushu University, Fukuoka, Fukuoka, Japan; 4 Forestry and Forest Products Research Institute, Tsukuba, Ibaraki, Japan; 5 Department of Neurological Science, Tohoku University Graduate School of Medicine, Sendai, Miyagi, Japan; Van Andel Institute, UNITED STATES

## Abstract

The global outbreak of bovine spongiform encephalopathy (BSE) has been attributed to the recycling of contaminated meat and bone meals (MBMs) as feed supplements. The use of MBMs has been prohibited in many countries; however, the development of a method for inactivating BSE prions could enable the efficient and safe use of these products as an organic resource. Subcritical water (SCW), which is water heated under pressure to maintain a liquid state at temperatures below the critical temperature (374°C), exhibits strong hydrolytic activity against organic compounds. In this study, we examined the residual *in vitro* seeding activity of protease-resistant prion protein (PrP^Sc^) and the infectivity of BSE prions after SCW treatments. Spinal cord homogenates prepared from BSE-infected cows were treated with SCW at 230–280°C for 5–7.5 min and used to intracerebrally inoculate transgenic mice overexpressing bovine prion protein. Serial protein misfolding cyclic amplification (sPMCA) analysis detected no PrP^Sc^ in the SCW-treated homogenates, and the mice treated with these samples survived for more than 700 days without any signs of disease. However, sPMCA analyses detected PrP^Sc^ accumulation in the brains of all inoculated mice. Furthermore, secondary passage mice, which inoculated with brain homogenates derived from a western blotting (WB)-positive primary passage mouse, died after an average of 240 days, similar to mice inoculated with untreated BSE-infected spinal cord homogenates. The PrP^Sc^ accumulation and vacuolation typically observed in the brains of BSE-infected mice were confirmed in these secondary passage mice, suggesting that the BSE prions maintained their infectivity after SCW treatment. One late-onset case, as well as asymptomatic but sPMCA-positive cases, were also recognized in secondary passage mice inoculated with brain homogenates from WB-negative but sPMCA-positive primary passage mice. These results indicated that SCW-mediated hydrolysis was insufficient to eliminate the infectivity of BSE prions under the conditions tested.

## Introduction

It is widely believed that protease-resistant, misfolded forms of prion proteins (PrP^Sc^) comprise the infectious agents that cause prion diseases such as Creutzfeldt-Jacob disease (CJD) in humans, scrapie in sheep and goats, chronic wasting disease (CWD) in deer and elk, and bovine spongiform encephalopathy (BSE) in cattle [1]. The first documented case of BSE occurred in the United Kingdom in 1986 [2]. PrP^Sc^ are highly resistant to conventional sterilization procedures [3], including physical [4, 5], chemical [6–9], and enzymatic treatments [10–13]. Thus, industrial processes for rendering of bovine slaughter offal to meat and bone meals (MBMs) are insufficient to completely inactivate prion infectivity [14, 15]. Because contaminated MBMs are suspected to be the source of BSE infection, the governments of many countries have prohibited the feeding of ruminant MBMs.

BSE prions are more resistant to inactivation treatments than scrapie and CJD prions [16]. Indeed, autoclaving at higher temperature (>155°C) [17], or heat-treatment in yellow grease at higher temperature and for longer time (180°C for 3 hours) [15] are required to inactivate the infectivity of BSE prions than scrapie prions. However, protein misfolding cyclic amplification (PMCA) analysis, a highly sensitive technique for detection of PrP^Sc^ [18], demonstrated that minute amounts of PrP^Sc^ remain after such treatments. Therefore, the development of an effective method for enabling the utilization of bovine MBMs as an organic resource would require thorough evaluation of both the levels of PrP^Sc^ and the residual infectivity of BSE prions.

Water that remains in a liquid state at temperatures of 100–374°C under pressurized conditions is referred to subcritical water (SCW). Although water is ionized via the collision of water molecules at ordinary temperatures and pressures, ionization progresses as the temperature increases. As a result, the concentration of hydroxide ions (OHˉ), which are capable of hydrolyzing the peptide bonds of proteins, as well as lipids and carbohydrates, to produce free amino acids [19], fatty acids [20], and glucose [21], respectively, is markedly higher in SCW than in normal water. Notably, the optimal hydrolytic activity of SCW occurs at approximately 250°C [22].

SCW has been shown to be effective for the hydrolysis and utilization of various natural organic materials, including food waste [23–25], sewage sludge [26], and waste wood [22, 27]. In addition, this method has numerous potential applications in the pharmaceutical industry [28]. The development of an SCW approach that effectively inactivates BSE prions would enable the usage of the valuable reaction products obtained from bovine MBMs including specific risk materials (SRMs), including brains, spinal cords, and tonsils [22, 29], instead of these materials being discarded as waste. Therefore, in this study, we evaluated the effects of SCW treatments on the residual seeding activity of PrP^Sc^ and on the infectivity of BSE prions.

## Materials and Methods

### Ethics Statement

All animal experiments were carried out in strict accordance with the regulations outlined in the Guide for the Care and Use of Laboratory Animals of the National Institute of Animal Health and the Guidelines for Proper Conduct of Animal Experiments (2006) by the Science Council of Japan. Procedures involving animals were approved by the Institutional Animal Care and Use Committee at the National Institute of Animal Health (approval ID: 08–008, 11–008, and 12–014), with all possible effort made to minimize the pain and discomfort of each animal in accordance with the Guidelines for Animal Transmissible Spongiform Encephalopathy Experiments of the Ministry of Agriculture, Forestry, and Fisheries of Japan. The animals were housed in a biosafety level three animal room, and intracerebral inoculation was performed under anesthesia. General anesthesia was achieved by administration of an inhalation anesthetic (5% sevoflurane). Their clinical status was monitored every 2 days. The animals were euthanized by anesthesia overdose following evidence of progressive neurological dysfunction such as slight tremor and depressive reaction or appearance of sings of aging such as weight loss and wasting.

### Subcritical Water Treatment

Spinal cords were obtained from four cows, which were experimentally inoculated with BSE intracerebrally, at the terminal stage of the disease [30]. The infectious materials were pooled, homogenized using a blender, and stored in small aliquots at –80°C. The spinal cord homogenate (SCH) was diluted by 20% (w/v) with distilled water, and the fibrous substances contained in the homogenate were solubilized by sonication for 20 min at 4°C. The 20% SCH (8 ml) was then added to an Inconel reactor tube (20 ml internal capacity) equipped with thermo and pressure sensors. The tubes were tightly sealed, and heated in a salt bath (Toyo Koatsu, Inc., Hiroshima, Japan) specially designed for this study. The entire reaction process, including the immersion of the tube in the pre-heated salt bath, sample agitation during the SCW treatment, and the rapid cooling of the tube in a water bath, was computer automated. The sample temperature and pressure were monitored in real time throughout the experimental process. Via a series of experiments using bovine MBMs, we determined that the majority of the organic compounds within the MBM were solubilized into water within 5 min when SCW treatments were performed above 230°C. Therefore, SCW treatments were performed under several conditions: at 230°C and 3.2 Mpa, 250°C and 4.5 MPa, and at 280°C and 7.1 MPa for 5 or 7.5 min, as listed in Table 1. Following treatment, 8 ml of 2× phosphate buffered saline (PBS) was added to the reactor tube, and the resulting solution (10% SCH) was recovered by pipetting. The aqueous fraction of the sample was then harvested by brief centrifugation, and stored at –80°C until further use. Immediately prior to use, samples were thawed and crushed using a mortar and pestle.

**Table 1:** Survival time of TgBoPrP mice following intracerebral inoculation of subcritical water (SCW)-treated bovine spongiform encephalopathy-infected spinal cord homogenates (BSE-SCH).

Inoculum	Condition	Mouse ID number	Transmission rate (diseased/total)	Survival time (average ± SD, days)	WB^d^ positive ratioosit	sPMCA^e^ positive ratio
BSE-SCH^a^	Untreated^b^		100% (7/7)	243 ± 14	100% (2/2)	NT
	280°C 7.5 min	1–7	0% (0/6)	>700	0% (0/6)	100% (6/6)
	280°C 5 min	7–11	0% (0/5)	>700	0% (0/5)	100% (5/5)
	250°C 7.5 min	12–17	0% (0/6)	>700	17% (1/6)	100% (6/6)
	250°C 5 min	18–22	0% (0/5)	>700	0% (0/5)	100% (5/5)
	230°C 7.5 min	23–28	0% (0/6)	>700	0% (0/6)	100% (6/6)
	230°C 5 min	29–34	0% (0/6)	>700	0% (0/6)	100% (6/6)
PBS^c^		35–40	0% (0/6)	>700	0% (0/6)	0% (0/6)

WB, western blot; sPMCA, serial protein misfolding cyclic amplification; NT, not tested; SD, standard deviation

^a^The infectious titer of the homogenate was estimated to be approximately 10^6.7^ LD_50_ per gram in our previous study [15].

^b^Mice inoculated with untreated SCH were used as a positive control.

^c^Mice inoculated with phosphate buffered saline (PBS) were used as a negative reaction.

^d^The results of Fig 2 are summarized in this column.

^e^The results of Fig 4 are summarized in this column.

### PMCA Analysis

In a previous study, we developed an ultrasensitive method for BSE PrP^Sc^ detection using potassium dextran sulfate (DSP) [31]. The PMCA method comprises an effective test for assessing prion inactivation by monitoring residual BSE PrP^Sc^ levels [15, 17]. Briefly, the brains of Tg(BoPrP)4092HOZ/Prnp0/0 transgenic (TgBoPrP) mice, which overexpress (approximately 8 times) bovine PrP^C^ [32], and PrP knockout (PrP^0/0^) mice were homogenized separately in PBS containing 1% Triton X-100 and 4 mM ethylenediaminetetraacetic acid (EDTA). After centrifugation at 4,500 × g for 5 min, the PrP^0/0^ and TgBoPrP supernatants were mixed at a 5:1 ratio, respectively. A mixture containing 0.5% DSP was used as the PrP^C^ substrate for PMCA analysis. The 10% SCH samples treated with SCW were mixed at 1:9 with the PrP^C^ substrate (total volume, 100 μl) in electron beam-irradiated polystyrene tubes. PMCA reactions were performed in duplicate using a fully automated cross-ultrasonic protein-activating apparatus (Elestein 070-CPR, Elekon Science, Chiba, Japan), which has the capacity to generate high levels of ultrasonic power (700 W). The amplification procedure consisted of 40 cycles of sonication (3 s pulse oscillations repeated 5 times at 1 s intervals), followed by incubation for 1 h at 37°C with agitation. For serial PMCA, 1:5 dilution of the PMCA product and subsequent amplification was repeated up to a maximum of four times. To detect PrP^Sc^ in the brains of the primary and secondary passage transgenic mice, 10% brain homogenates (BH) were mixed 1:9 with the PrP^C^ substrate (total volume, 80 μl) and amplified in electron beam-irradiated eight-strip polystyrene tubes (076–96, Elekon Science). Amplification [40 cycles of sonication (pulse oscillation for 5 s, repeated 5 times at 1 s intervals), followed by incubation for 1 h at 37°C with agitation] was performed in quadruplicate. For serial PMCA analysis, 1:5 dilution of the amplified product and subsequent amplification was repeated 3 times.

### Western Blotting

PMCA samples (10 μl) were incubated with 10 μl proteinase K solution (100 μg/ml) at 37°C for 1 h, and then mixed with 20 μl of 2× sodium dodecyl sulfate (SDS) sample buffer and incubated at 100°C for 5 min. The samples were separated by SDS-polyacrylamide gel electrophoresis (PAGE) and transferred to polyvinylidene fluoride (PVDF) membranes (Millipore, Billerica, MA, USA). After blocking, the membranes were incubated for 30 min with a horseradish peroxidase (HRP)-conjugated T2 monoclonal antibody [33], which recognizes a discontinuous epitope comprised by amino acid residues 132–156 of the mouse PrP sequence, and has also been shown to react with bovine PrP [34]. Membranes were then washed and developed using the Luminata Forte Western HRP Substrate (Millipore), according to the manufacturer’s instructions. Chemiluminescence signals were detected using a Light Capture System (Atto, Tokyo, Japan).

### Bioassay

The SCW-treated bovine spinal cord samples were injected intracerebrally into five or six TgBoPrP mice (20 μl per mouse). To examine the infectivity of the PrP^Sc^ present in the primary passage mice, 10% BH of several western blot (WB)-positive or sPMCA-positive mice were subsequently injected intracerebrally into four to seven TgBoPrP mice (20 μl per mouse). After inoculation, the mice were evaluated daily for signs of disease.

### Histological and Immunohistochemical Analysis

The left hemispheres of mouse brains were fixed in 10% buffered formalin for subsequent neuropathological analyses. Coronal slices of the brain were immersed in 98% formic acid to reduce the infectivity, and embedded in paraffin wax. Sections (4 μm thickness) were cut and stained with hematoxylin and eosin, and subjected to immunohistochemical analysis using with SAF84 monoclonal antibody (SPI-bio, Montigny le Bretonneux, France), as described previously [35].

## Results

### Detection of PrP^Sc^ in the SCW-Treated SCH

**Fig 1:**
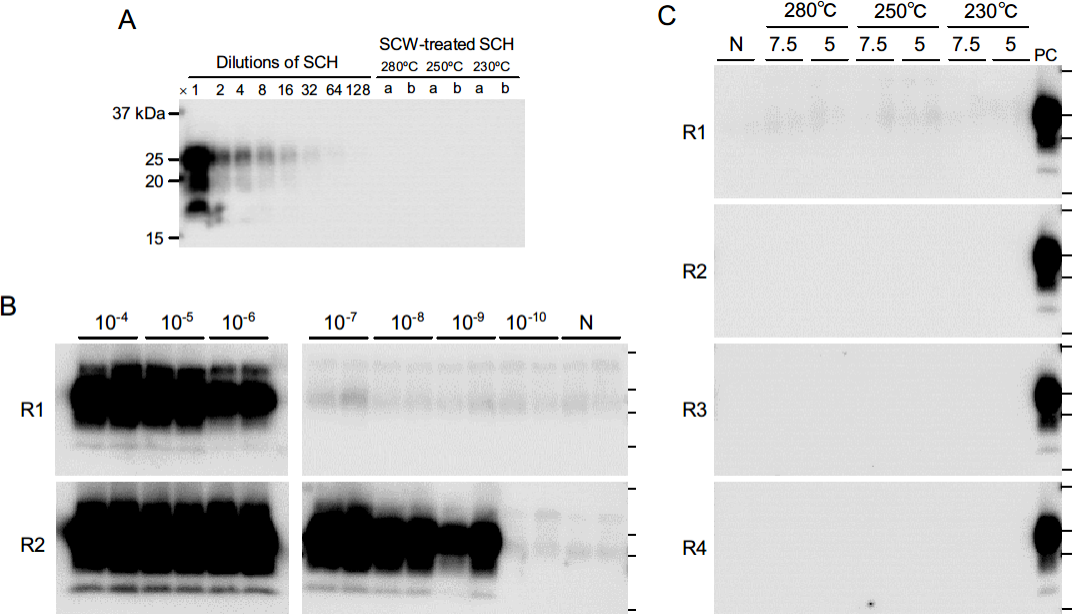
Western blot (WB) analysis of bovine spongiform encephalopathy (BSE)-infected spinal cord homogenates (BSE-SCH) before and after subcritical water (SCW) treatment. (**A**) A 10% BSE-SCH were serially diluted (1- to 128-fold), digested with proteinase K (PK), and analyzed by WB. PrP^Sc^ signals were recognized in the samples diluted up to 32-fold. BSE-SCH samples treated with SCW at 230°C, 250°C, or 280°C for 7.5 (a) or 5 min (b) were analyzed by WB after digestion with PK. (**B**) Amplification of PrP^Sc^ in diluted BSE-SCH samples. A 10% BSE-SCH was diluted (10^−4^–10^−10^) and serially amplified by protein misfolding cyclic amplification (PMCA). After each round (R1–R4) of amplification, duplicate samples were digested with PK and analyzed by WB. The lanes labeled “N” indicate samples in which the PrP^C^ substrate alone was treated in the same manner. (**C**) PMCA analysis of PrP^Sc^ in SCW-treated samples. BSE-SCH were treated with SCW at 230°C, 250°C, or 280°C for 7.5 or 5 min, and serially amplified by PMCA. Duplicate samples were analyzed after each round (R1–R4) of amplification. The lane labeled “PC” indicates the positive control sample containing untreated BSE-SCH diluted to 10^−4^. Horizontal lines indicate the positions of the 37, 25, 20, and 15 kilodalton (kDa) bands of the molecular-weight marker.

We first assessed whether there were residual PrP^Sc^ present in the SCH samples subjected to the SCW treatments listed in Table 1. No PrP^Sc^ signal was detected by conventional WB analysis in any of the SCW-treated samples (Fig 1A), indicating that such treatments were sufficient to alter or break down the T2 epitope of the prion protein. In a previous study, we demonstrated that PrP^Sc^ derived from BSE-infected cattle could be efficiently amplified by sPMCA in the presence of DSP [31]. We therefore utilized this highly sensitive method to determine whether there was residual PrP^Sc^ in the SCW-treated SCH samples. PrP^Sc^ were amplified from the untreated BSE-infected SCH (10%) samples diluted up to 10^−6^ and10^-9^ after one and two rounds of amplification, respectively (Fig 1B). On the other hand, no PrP^Sc^ signal was detected in the SCW-treated samples, even after four rounds of amplification (Fig 1C), suggesting that the SCW treatments effectively abrogated the *in vitro* seeding activity of the BSE PrP^Sc^.

### Infectivity of the SCW-Treated SCH

**Fig 2:**
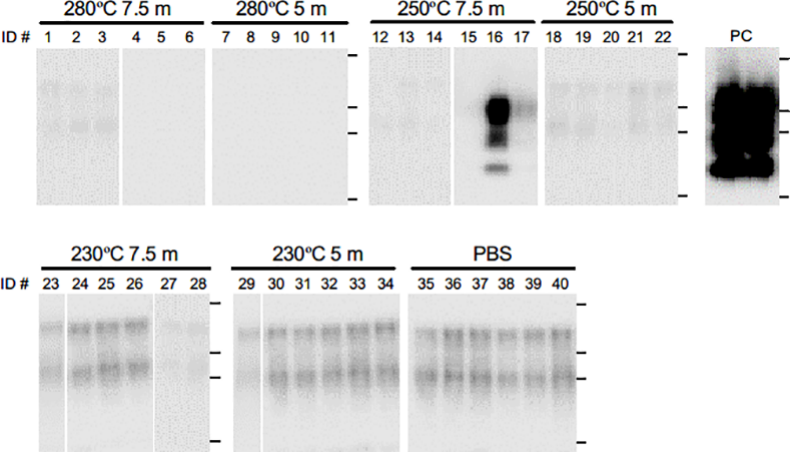
Western blot (WB) analysis of brains of mice inoculated intracerebrally with subcritical water (SCW)-treated bovine spongiform encephalopathy (BSE)-infected spinal cord homogenates (BSE-SCH). BSE-SCH were treated with SCW at 230°C, 250°C, or 280°C for 7.5 min or 5 min. A 10% brain homogenate was prepared from each mouse, digested with proteinase K, and subjected to WB analysis. Mice inoculated with PBS were used as a negative reaction and mice inoculated with diluted untreated BSE-SCH (PC) were used as positive controls. As seen in the mice inoculated with PBS, extra bands with a molecular weight higher than that for PrP^Sc^ were occasionally observed, likely corresponding to prion protein aggregates or to residue of the normal isoform of prion protein resulting from incomplete PK digestion. Horizontal lines indicate the positions of the 37, 25, 20, and 15 kilodalton (kDa) bands of the molecular-weight marker.

**Fig 3:**
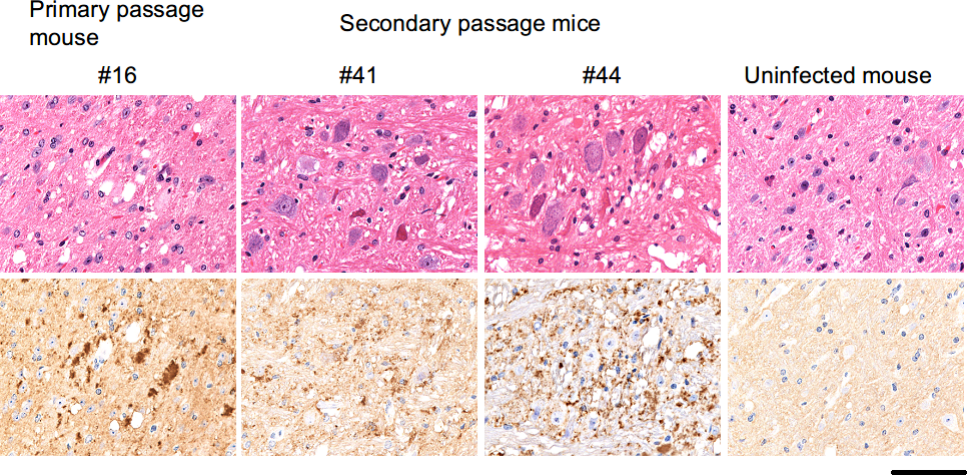
Histological and immunohistochemical analysis of the primary and secondary passage mice. A section of the pontine nucleus of the western blot-positive but asymptomatic primary passage mouse (ID #16) showing vacuolation (upper panel, hematoxylin and eosin staining) and PrP^Sc^ accumulation (lower panel, labelled with SAF84 antibody). Similar lesions were observed in the brains of diseased secondary passage mice (ID #41 and 44). No vacuolation or PrP^Sc^ accumulation was observed in uninfected mouse. Bars, 50 μm.

The results of the bioassay analyses using TgBoPrP mice are summarized in Table 1. The mice inoculated with the 10% solution of untreated SCH died after an average period of 243 days. On the other hand, none of the mice inoculated with the SCW-treated SCH developed the disease throughout the observation tested (>700 days). To evaluate the accumulation of PrP^Sc^ in these animals, brains were subjected to analysis. WB analysis detected the three protease-resistant bands typical of BSE in one (Fig 2, ID #16) of the six mice inoculated with the SCH subjected to SCW at 250°C for 7.5 min. In addition, although the #16 mouse was physically asymptomatic, the pontine nucleus of this animal exhibited typical lesions of prion diseases, such as vacuolation and PrP^Sc^ accumulation (Fig 3, ID #16), which were not present in the brain of the uninfected mouse. Indeed, this mouse was considered to be at one stage of subclinical infection [36–38]. These results indicate that BSE prions from the SCH treated with 250°C SCW for 7.5 min remained infectious despite a lack of *in vitro* PrP^Sc^ seeding activity.

### Detection of PrP^Sc^ in the Brains of the Primary Passage Mice by sPMCA

**Fig 4:**
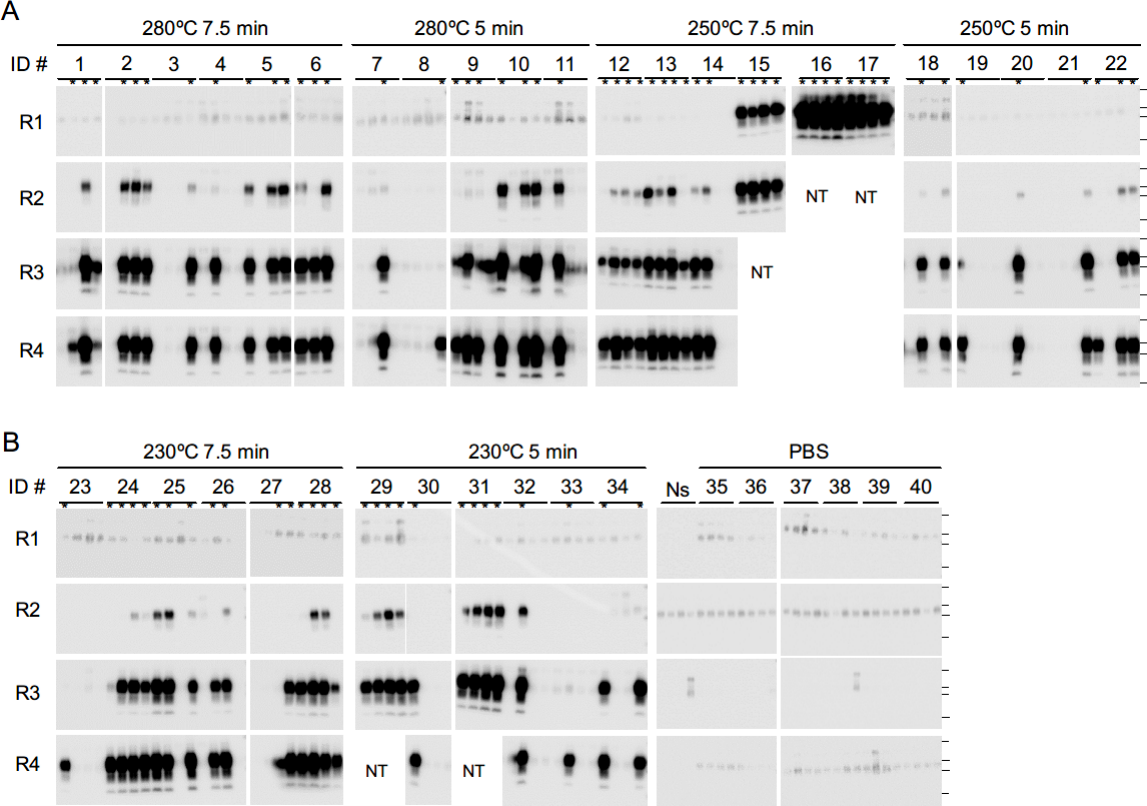
PrP^Sc^ detection in brains of asymptomatic mice inoculated with the subcritical water (SCW)-treated bovine spongiform encephalopathy (BSE)-infected spinal cord homogenates (BSE-SCH). (**A**) 10% brain homogenates were prepared from the primary passage mice intracerebrally inoculated with samples treated at 280°C for 7.5 min (ID #1–6) or 5 min (ID #7–11), at 250°C for 7.5 min (ID #12–17) or 5 min (ID #18–22), and (**B**) at 230°C for 7.5 min (ID #23–28) or 5 min (ID #29–34), and amplified by serial protein misfolding cyclic amplification (sPMCA). Mice inoculated with phosphate buffered saline (ID #35–40) were used as a negative reaction. After each round (R1–R4) of amplification, quadruplicate samples were digested with proteinase K and analyzed by western blot. The four lanes labeled “Ns” indicate samples in which the PrP^C^ substrate alone was treated. Horizontal lines indicate the positions of the 37, 25, 20, and 15 kilodalton (kDa) bands of the molecular-weight marker. The positive lanes are denoted by asterisk symbol on the top of the blots. NT, not tested.

To determine whether there were infinitesimal amounts of PrP^Sc^ in the brains of the primary passage mice, sPMCA was conducted using the BH of inoculated mice as a seed. Of the mice inoculated with the SCH treated at 250°C for 7.5 min, in addition to ID #16, two (Fig 4A, ID #15 and #17) tested positive for PrP^Sc^ in each of the quadruplicate samples after one round of amplification. While PrP^Sc^ were also detected in the other three mice in this group (ID #12–14) after two or three rounds of sPMCA, the ratio of positive tests in the quadruplicate samples varied among these mice (50–100%) after four rounds of sPMCA. Additionally, PrP^Sc^ were detected in each of the mice inoculated with SCH treated at 250°C for 5 min (Fig 4A, ID #18–22) after four rounds of sPMCA, with positive test ratios between 25–75%. Similarly, all mice inoculated with the SCH treated at 280°C (Fig 4A, ID #1–11) or 230°C for 5–7.5 min (Fig 4B, ID #23–34) were PrP^Sc^-positive within four rounds of sPMCA, with positive ratios between 25–100%. In contrast, no PrP^Sc^ signal was detected in the mice inoculated with PBS (Fig 4B, ID #35–40; Table 1), suggesting that the appearance of PrP^Sc^ was not simply the result of aging. Together, these results indicate that the infectivity of the BSE prions were not completely eliminated by the SCW treatments evaluated in this study.

### Analysis of the SCW-Treated BSE Prion Infectivity by Serial Transmission

**Fig 5:**
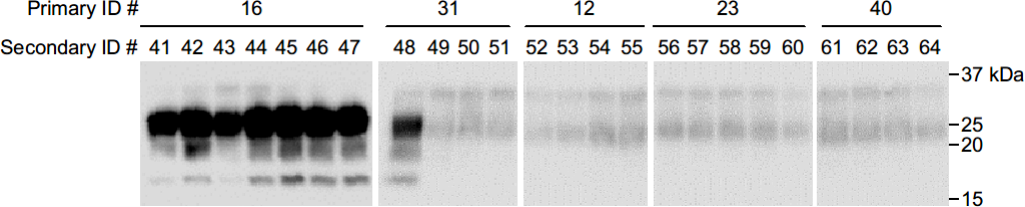
Western blot (WB) analysis of the brains of the secondary passage mice. 10% brain homogenates were prepared from the secondary passage mice (secondary ID #41–64) inoculated intracerebrally with the brain homogenates derived from the WB-positive mouse (primary ID #16), serial protein misfolding cyclic amplification (sPMCA)-positive mice (primary ID #31, 12 and 23), and analyzed by WB after digestion with proteinase K. The PBS-inoculated mouse (primary ID #40) was used as a negative reaction. Horizontal lines indicate the positions of the 37, 25, 20, and 15 kilodalton (kDa) bands of the molecular-weight marker.

The above results implied that mice inoculated with SCW-treated SCH likely accumulate PrP^Sc^ in the brains, but that the onset of disease might not occur within their lifetime. To examine the potential pathogenicity of the SCW-treated BSE prions, BHs prepared from primary passage mice exhibiting distinct levels of PrP^Sc^ accumulation were serially transmitted into TgBoPrP mice (Table 2). The secondary passage mice inoculated with BH from the conventional WB-positive but asymptomatic mouse (ID #16) presented clinical signs of the disease, including tremors and weight loss, and all mice died after an average period of 240 days. There was no significant difference between this survival period and that of the mice inoculated with the untreated SCH (Table 1). Moreover, the brains of these seven mice were positive for PrP^Sc^ by WB (Fig 5, secondary ID #41–47), and immunohistochemical analysis detected typical lesions of prion diseases in each of the two brains examined (Fig 3, secondary ID #41 and 44). Therefore, the pathogenicity of the BSE prion remained despite the SCW treatment.

**Fig 6:**
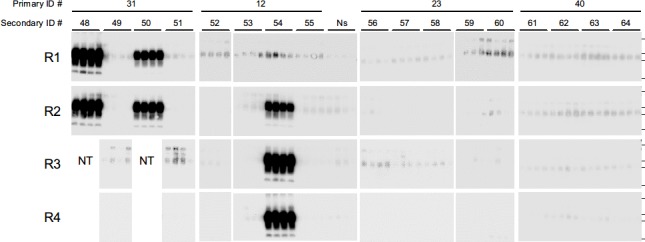
Detection of PrP^Sc^ in the brains of the secondary passage mice. 10% brain homogenates were prepared from the secondary passage mice (secondary ID #48–64) inoculated intracerebrally with the brain homogenates derived from the serial protein misfolding cyclic amplification (sPMCA)-positive mice (primary ID #31, 12 and 23), and amplified by sPMCA. After each round (R1–R4) of amplification, quadruplicate samples were digested with proteinase K and analyzed by western blotting. The PBS-inoculated mouse (primary ID #40) was used as a negative reaction. The four lanes labeled “Ns” indicate samples in which the PrP^C^ substrate alone was treated. Horizontal lines indicate the positions of the 37, 25, 20, and 15 kilodalton (kDa) bands of the molecular-weight marker. NT, not tested.

**Table 2:** Survival time of the secondary passage TgBoPrP mice.

Mouse ID number in the primary transmission study	WB^a^	sPMCA^b^ (positive ratio)	Transmission rate (diseased/total)(diseased/total)	Survival time (days)
16	+	R1 (100%)	100% (7/7)	206, 210, 212, 253, 263, 263, 270
31	-	R2 (100%)	25% (1/4)	601^c^, 799, 809^d^, 810
12	-	R3 (100%)	0% (0/4)	611, 770, 793^d^, 826
23	-	R4 (25%)	0% (0/5)	756, 820, 854, 856, 856
40	-	R4 (0%)	0% (0/4)	621, 718, 733, 733

WB, western blot; sPMCA, serial protein misfolding cyclic amplification

^a^Results of conventional WB analysis of 10% brain homogenates of the indicated mice (Fig 2). A typical PrP^Sc^ signal was detected in mouse #16.

^b^Results of sPMCA analysis of 10% brain homogenates of the indicated mice (Fig 4). Number(s) of rounds of amplification (R1–R4) needed to detect PrP^Sc^ signal at the indicated positive ratio are shown.

^c^Mouse that exhibited typical clinical signs of prion diseases, such as tremors and wasting, and for which the accumulation of PrP^Sc^ within the brain was confirmed by conventional WB analysis (Fig 5, secondary ID #48).

^d^While no clinical signs of prion disease were observed, PrP^Sc^ were detected in the brains of these mice by sPMCA (Fig 6, secondary ID #50 and 54).

One of the mice subjected to serial transmission of the BH from mouse #31, which tested positive for PrP^Sc^ after two rounds of sPMCA, exhibited disease onset at 601 days post-inoculation (Table 2), and PrP^Sc^ accumulation was confirmed in the brain of this mouse by conventional WB analysis (Fig 5, secondary ID #48). In addition, PrP^Sc^ were detected in the brain of another mouse (Fig 6, secondary ID #50) after one round of amplification; however, this mouse remained healthy during the observation period. Similarly, while none of the mice inoculated with BH from mouse #12, which tested positive for PrP^Sc^ after three rounds of sPMCA, developed the disease (Table 2), PrP^Sc^ were detected in one of the mice (Fig 6, secondary ID #54) after two rounds of sPMCA. Lastly, each of the mice inoculated with the BH of mouse #23, which tested positive for PrP^Sc^ after four rounds of sPMCA, with a positive ratio of 25%, survived for more than 750 days without any signs of the disease (Table 2), and no PrP^Sc^ was found in the brains of these animals by sPMCA analysis (Fig 6, secondary ID #56–60).

## Discussion

In the present study, we examined the practical effectiveness of SCW-mediated hydrolysis for inactivation of BSE prions, in terms of residual *in vitro* seeding activity and infectivity. No significant seeding activity of PrP^Sc^ was detected in the SCW-treated SCH, and all mice inoculated intracerebrally with the SCW-treated SCH survived for more than 700 days with no disease symptoms. However, PrP^Sc^ accumulation was detected in the brain of a mouse inoculated with the SCH treated at 250°C for 7.5 min by WB analysis. In addition, PMCA analysis detected PrP^Sc^ in the brains of all inoculated mice. Furthermore, early and late-disease onset cases were recognized in the secondary passage mice inoculated with BH derived from the primary passage mice. These observations indicated that the SCW hydrolysis procedures examined in this study were ineffective for completely eliminating the infectivity of BSE prions.

Previously, we demonstrated that PrP^Sc^ seeding activity is typically observed in samples in which infectivity was detected using bioassays [15, 17, 39]. However, in the present study, low levels of infectivity remained in the BSE-infected SCH after the SCW treatments, despite the observed lack of PrP^Sc^ seeding activity. There was no apparent correlation between the PrP^Sc^ content of the brains of the primary passage mice and the SCW conditions (temperature or time) used for inactivation of BSE-SCH. While relatively higher levels of PrP^Sc^ accumulation were detected in three primary passage mice (ID #16 in Fig 2, and ID #15 and #17 in Fig 4) inoculated with BSE-SCH treated with 250°C SCW for 7.5 min, seeding activity was not observed in the other 80 samples subjected to this treatment (S1 Fig 1).

As expected, infectivity was detected in the secondary passage mice inoculated with BH derived from the primary passage mice exhibiting varying PrP^Sc^ levels when PrP^Sc^ were present in the inocula (Fig 2 and Table 2, ID #16; Fig 4 and Table 2, ID #31). Therefore, the lack of seeding activity of the SCW-treated samples is likely attributable to the physical and chemical characteristics of SCW. The SCW treatment process can promote various chemical reactions, including hydrolysis, oxidation, and dehydration. As such, one possible explanation for our findings is that PrP^Sc^ amplification might be inhibited by certain chemical compounds generated by the SCW treatment. Indeed, WB analyses detected a reduction in the signal intensity of PrP^Sc^ upon the addition of the SCW-treated samples to the amplification reaction (S2 Fig). Therefore, inhibitory agents produced within the SCW-treated samples may contribute to the reduced seeding activity of PrP^Sc^.

One plausible explanation is that the active site required for the expression of seeding activity is degraded during the SCW treatment, while regions that are essential for infectivity are more resistant to the treatments and are therefore not completely decomposed. To address this possibility, we conducted a series of experiments using bovine MBMs to define the optimal SCW conditions for achieving hydrolysis of organic compounds. At 230–250°C, solubilized proteins were hydrolyzed in a time-dependent manner; however, the maximum yield of amino acids was achieved after treatment for 5 min at 280°C. Meanwhile, the amino acid recovery rate was usually estimated to be below 20% of the total amount of nitrogen contained in MBM. We therefore concluded that not all proteins were completely hydrolyzed into amino acids, and that significant amounts of protein remained as peptides under the experimental conditions tested. Indeed, gel permeation chromatography analysis indicated that the SCW-treated samples contained considerable amounts of peptides weighing between 1,000 and 3,000 Daltons (Da; unpublished work). Such undecomposed BSE PrP^Sc^ residues might serve as minimal infectious units that are capable of inducing subclinical infections in inoculated mice.

In conclusion, we demonstrated that SCW treatments suitable for recycle use of MBM failed to completely inactivate BSE prions. Although prion inactivation may proceed more effectively at higher temperature regions around supercritical water or in the presence of longer heating times, such harsh treatments would cause severe damage to the valuable organic materials present in the MBM, resulting in reduced economic merits.

## Supporting Information

S1 Fig Protein misfolding cyclic amplification (PMCA) analysis of samples treated with subcritical water (SCW) at 250°C for 7.5 min. Bovine spongiform encephalopathy (BSE)-infected spinal cord homogenates **(**SCH) were treated with SCW at 250°C for 7.5 min, and serially amplified by PMCA. After each round (R1–R4) of amplification, 80 samples (lane 1–80) were digested with proteinase K and subjected to western blot analysis. The lanes labeled “PC” indicate the positive control samples, which contained untreated BSE-SCH diluted from 10^−6^ to 10^−9^. The lanes labeled “Ns” indicate samples in which only the PrP^C^ substrate was treated in the same manner.
(TIF)

S2 Fig Inhibitory effect of subcritical water (SCW)-treated samples on protein misfolding cyclic amplification (PMCA). Bovine spongiform encephalopathy (BSE)-infected spinal cord homogenates **(**SCH) (10%) were diluted to 10^−4^, and amplified in the presence of 1–8 μl of BSE-SCH treated with SCW at 250°C for 7.5 min or PBS. After amplification, the samples were digested with proteinase K and analyzed by western blot. Densitometric analysis indicated a reduction in the PrP^Sc^ signal intensity to 84% (1 μl), 72% (2 μl), 68% (4 μl), and 62% (8 μl) of that of the respective PBS-added samples after the addition of the SCW-treated product.
(TIF)
