# Supplementary figures and images for: Subcritical Water Hydrolysis Effectively Reduces the In Vitro Seeding Activity of PrPSc but Fails to Inactivate the Infectivity of Bovine Spongiform Encephalopathy Prions
Source: PLoS One. 2015 Dec 16;10(12):e0144761. doi: 10.1371/journal.pone.0144761 (PMC4682654; doi:10.1371/journal.pone.0144761)

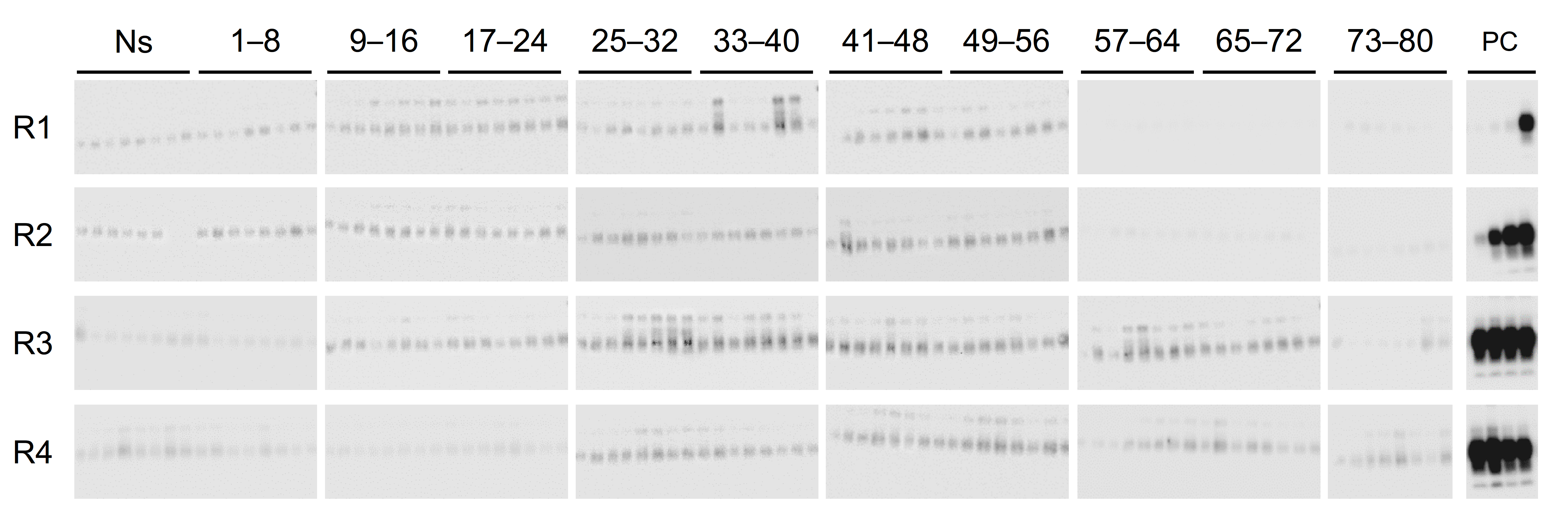

Supplement: S1 Fig — Bovine spongiform encephalopathy (BSE)-infected spinal cord homogenates (SCH) were treated with SCW at 250°C for 7.5 min, and serially amplified by PMCA. After each round (R1–R4) of amplification, 80 samples (lane 1–80) were digested with proteinase K and subjected to western blot analysis. The lanes labeled “PC” indicate the positive control samples, which contained untreated BSE-SCH diluted from 10−6 to 10−9. The lanes labeled “Ns” indicate samples in which only the PrPC substrate was treated in the same manner. (TIF) [file pone.0144761.s001.tif]

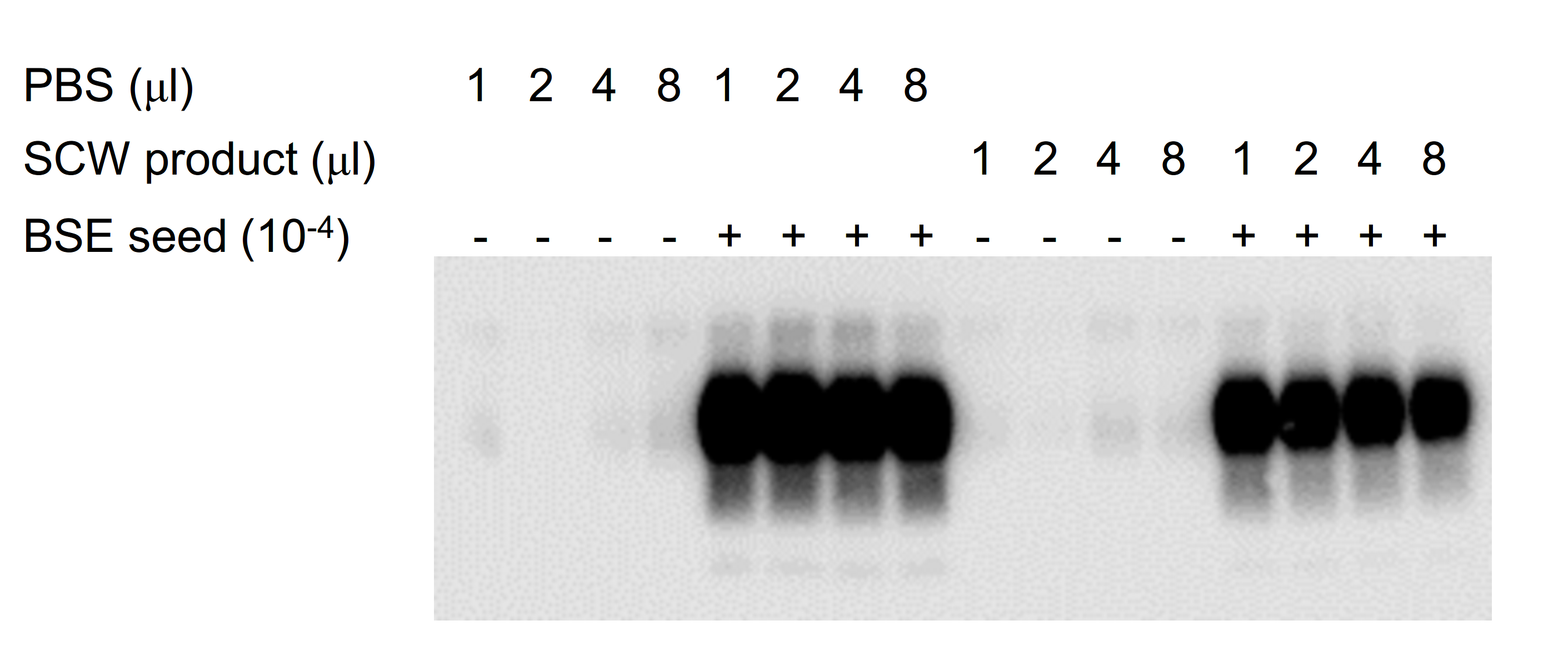

Supplement: S2 Fig — Bovine spongiform encephalopathy (BSE)-infected spinal cord homogenates (SCH) (10%) were diluted to 10−4, and amplified in the presence of 1–8 μl of BSE-SCH treated with SCW at 250°C for 7.5 min or PBS. After amplification, the samples were digested with proteinase K and analyzed by western blot. Densitometric analysis indicated a reduction in the PrPSc signal intensity to 84% (1 μl), 72% (2 μl), 68% (4 μl), and 62% (8 μl) of that of the respective PBS-added samples after the addition of the SCW-treated product. (TIF) [file pone.0144761.s002.tif]
